# A Camera-Based Target Detection and Positioning UAV System for Search and Rescue (SAR) Purposes

**DOI:** 10.3390/s16111778

**Published:** 2016-10-25

**Authors:** Jingxuan Sun, Boyang Li, Yifan Jiang, Chih-yung Wen

**Affiliations:** Department of Mechanical Engineering, The Hong Kong Polytechnic University, Hong Kong, China; jingxuan.j.sun@connecy.polyu.hk (J.S.); boyang.li@connect.polyu.hk (B.L.); jiang.uhrmacher@connect.polyu.hk (Y.J.)

**Keywords:** unmanned aerial vehicle (UAV), wilderness search and rescue, target detection

## Abstract

Wilderness search and rescue entails performing a wide-range of work in complex environments and large regions. Given the concerns inherent in large regions due to limited rescue distribution, unmanned aerial vehicle (UAV)-based frameworks are a promising platform for providing aerial imaging. In recent years, technological advances in areas such as micro-technology, sensors and navigation have influenced the various applications of UAVs. In this study, an all-in-one camera-based target detection and positioning system is developed and integrated into a fully autonomous fixed-wing UAV. The system presented in this paper is capable of on-board, real-time target identification, post-target identification and location and aerial image collection for further mapping applications. Its performance is examined using several simulated search and rescue missions, and the test results demonstrate its reliability and efficiency.

## 1. Introduction

Wilderness search and rescue (SAR) is challenging, as it involves searching large areas with complex terrain for a limited time. Common wilderness search and rescue missions include searching and rescuing injured humans and finding broken and lost cars in deserts, forests or mountains. Incidents of commercial aircraft disappearing from radar, such as the case in Indonesia in 2014 [1,2,3], also entail a huge search radius and search timeliness is critical to “the probability of finding and successfully aiding the victim” [4,5,6,7]. This research focuses on applications common in eastern Asian locations such as Hong Kong, Taiwan, the southeastern provinces of mainland China, Japan and the Philippines, where typhoons and earthquakes happen a few times annually, causing landslides and river flooding that result in significant damage to houses, roads and human lives. Immediate assessment of the degree of damage and searching for survivors are critical requirements for constructing a rescue and revival plan. UAV-based remote image sensing can play an important role in large-scale SAR missions [4,5,6,8,9].

With the development of micro-electro-mechanical system (MEMS) sensors, the use of small UAVs (with a wing-span of under 10 m) is a promising platform for conducting search, rescue and environmental surveillance missions. UAVs can be equipped with various remote sensing systems, such as powerful tools for observing disaster mitigation, including rapid all-weather flood and earthquake damage assessment. Today, low price drones allow people to quickly develop small UAVs, which have the following specific advantages:

- Can loiter for lengthy periods at preferred altitudes;
- Produce remote sensor data with better resolution than satellites, particularly in terms of image quality;
- Low cost, rapid response;
- Capable of flying below normal air traffic height;
- Can get closer to areas of interest.

Applying UAV technology and remote sensing to search, rescue and environmental surveillance is not a new idea. Habib et al. stated the advantages of applying UAV technologies to surveillance, security and mission planning, compared with the normal use of satellites, and various technologies and applications have been integrated and tested on UAV-assisted operations [9,10,11,12,13].

A fact people cannot ignore when applying UAV-assisted SAR is the number of required operators. It is claimed that at least two roles are required: one pilot who flies, monitors, plans and controls the UAV, and a second pilot who operates the sensors and information flow [14]. Practically, these two roles can be filled by a single operator, yet studies on ground robots have also suggested that a third person is recommended to monitor and protect the operator(s). Researchers have also studied the human behavior involved in managing multi UAVs, and have found that “the span of the human control is limited” [4,14,15]. As a result, a critical challenge of applying multiple UAVs in SAR is simultaneously monitoring information-rich data streams, including flight data and aerial video. The possibility of simplifying the human roles by optimizing information presentation and automatizing information acquisition was also explored [4], in which a fixed-wing UAV was used as a platform, and they analyzed and compared three computer vision algorithms to improve the presentation.

To automatize the information acquisition, it has been suggested that UAV systems integrate target-detection technologies for detecting people, cars or aircraft. A common method of observing people is the detection of heat features, which can be achieved by applying infrared camera technology and specifically developed algorithms. In 2005, a two-stage method based on a generalized template was presented [16]. In the first stage, a fast screening procedure is conducted to locate the potential person. Then, the hypothesized location of the person is examined by an ensemble classifier. In contrast, human detection based on color imagery has also been studied for many years. The research on developing a human detection method was conducted, which uses background subtraction, but pre-processing is required before a search mission [17]. Another method of human detection was presented that uses color images and models the human/flexible parts, then detects the parts separately [18]. A combination of both thermal and color imagery for human detection was also studied in [19].

To enhance information presentation and support humanitarian action, geo-referenced data from disaster-affected areas is expected to be produced. Numerous different technologies and algorithms for generating geo-referenced data via UAV have been studied and developed. A self-adaptive, image-matching technique to process UAV video in real-time for quick natural disaster response was presented in [20]. A prototype UAV and a geographical information system (GIS) by applying the stereo-matching method to construct a three-dimensional hazard map was also developed [21]. Scale Invariant Features Transform (SIFT) algorithms was improved in [22] by applying a simplified Forstner operator. Rectifying images on pseudo center points of auxiliary data were proposed in [23].

The aim of this study is to build an all-in-one camera-based target detection and positioning system that integrates the necessary remote sensors for wilderness SAR missions into a fixed-wing UAV. Identification and search algorithms were also developed. The UAV system can autonomously conduct a mission, including auto-takeoff and auto-landing. The on-board searching algorithm can report victims or cars with GPS coordinates in real-time. After the mission, a map of the hazard area can be generated to facilitate further logistics decisions and rescue troop action. Despite their importance, the algorithms for producing the hazard map are beyond the scope of this paper. In this work, we focus on the possibility of using a UAV to simultaneously collect geo-referenced data and detect victims. A hazard map and points are generated by the commercial software Pix4Dmapper^TM^ (Pix4Dmapper Discovery version 2.0.83, Pix4D SA, Lausanne, Switzerland).

**Figure 1:**
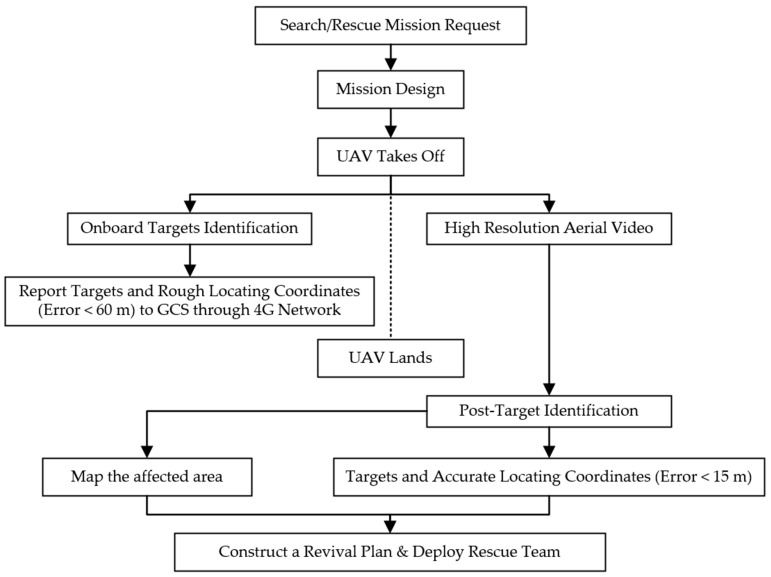
Flowchart of a wilderness SAR mission using the all-in-one UAV.

Figure 1 provides a mission flowchart. Once a wilderness SAR mission is requested to the Ground Control System (GCS), the GCS operator designs a flight path that covers the search area and sends the UAV into the air to conduct the mission. During the flight, the on-board image processing system is designed to identify targets such as cars or victims, and to report possible targets with the corresponding GPS coordinates to the GCS within 60 m accuracy. These real-time images and generalized GPS help the immediate rescue action including directing the victim to wait for rescue at the current location and delivering emergency medicine, food and water. Meanwhile, the UAV is transmitting real-time video to the GCS and recording high-resolution aerial video that can be used, once the UAV lands, in post-processing tasks such as target identification and mapping the affected area. The post-target identification is designed to report victims’ accurate locations within 15 m, and the map of the affected area can be used to construct a rescue plan.

The remainder of this paper is organized as follows. Section 2 describes the details of the UAV system. Section 3 presents the algorithm and the implementation. Section 4 presents the tests and results, and Section 5 concludes the paper.

## 2. Experimental Design

The all-in-one camera-based target detection and positioning UAV system integrates the UAV platform, the communication system, the image system, and the GCS. The detailed hardware construction of the UAV is introduced in this section.

### 2.1. System Architecture

The purpose of the UAV system developed in this study was to find targets’ GPS coordinates within a limited amount of time. To achieve this, a suitable type of aircraft frame was needed. The aircraft had to have enough fuselage space to accommodate the necessary payload for the task. The vehicle configuration and material had to exhibit the good aerodynamic performance and reliable structural strength needed for long-range missions. The propulsion system for the aircraft was calculated and selected once the UAV’s configuration and requirements were known.

**Figure 2:**
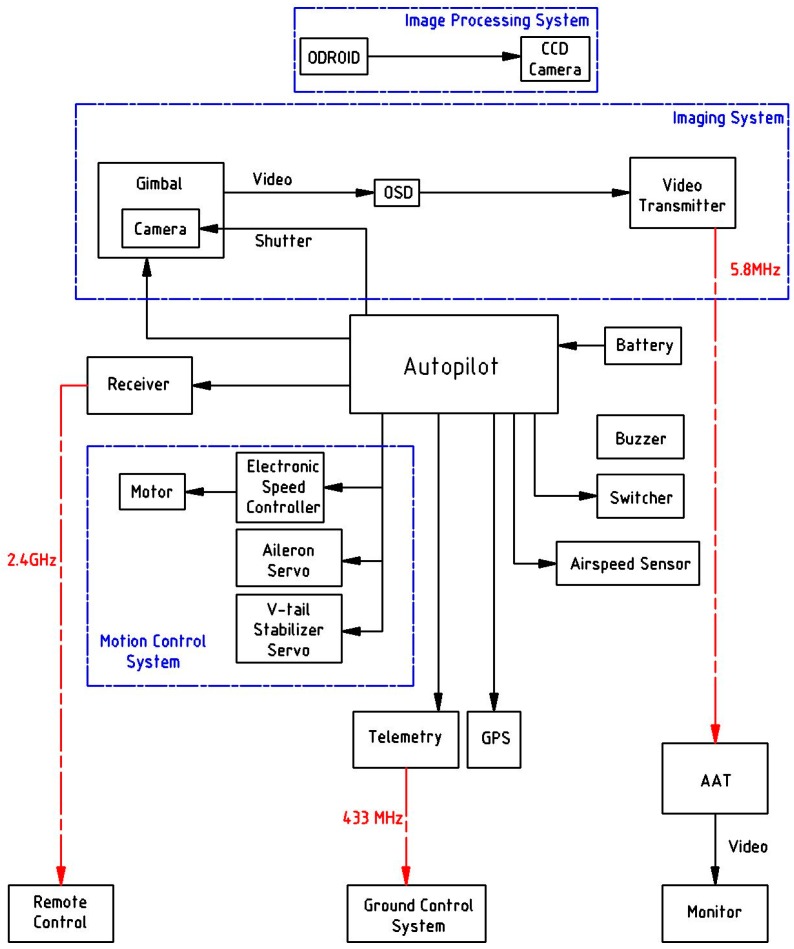
Systematic framework of the UAV system.

Next, a communication system, including a telemetry system, was used to connect the ground station to the UAV. After adding the flight control system, the aircraft could take off and follow the designed route autonomously. Finally, with the help of the mission system (auto antenna tracker (AAT), cameras, on-board processing board Odroid and gimbal), targets’ and their GPS coordinates could be found. Figure 2 shows the UAV system’s systematic framework, the details of which are explained in the following sub-sections. The whole system weighs 3.35 kg and takes off via hand launching.

### 2.2. Airframe of the UAV System

**Figure 3:**
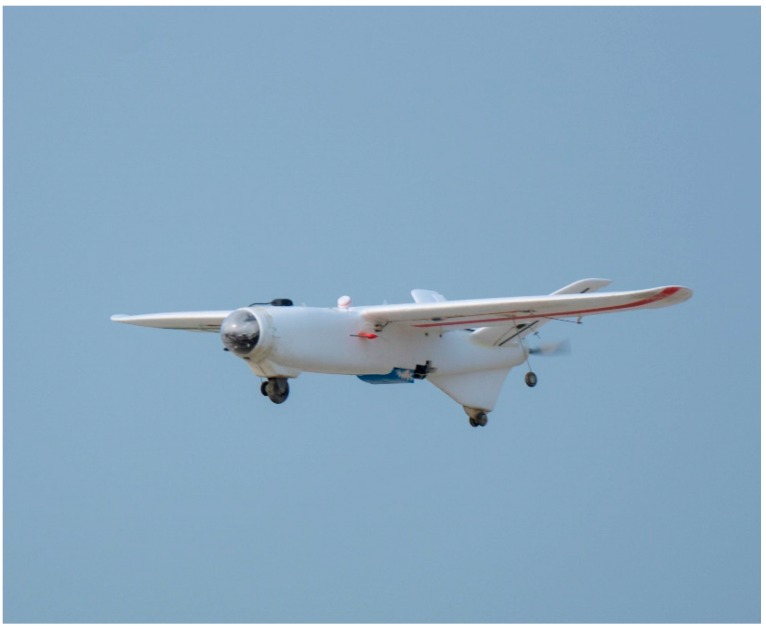
Overall View of X-UAV Talon [24].

The project objective was to develop a highly integrated system capable of large-area SAR missions. Thus, the flight vehicle, as the basic platform of the whole system, was chosen first. Given the prerequisites of quick response and immediate assessment capabilities, a fixed-wing aircraft was chosen for its high speed cruising ability, long range and flexibility in complex climatic conditions. To shorten the development cycle and improve system maintenance, an off-the-shelf commercial UAV platform “Talon” from X-UAV company was used (Figure 3). The wingspan of the Talon is 1718 mm and the wing area is 0.06 m^2^. The take-off weight of this airframe can reach 3.8 kg.

### 2.3. Propulsion System

The UAV uses Sunnysky X-2820-5 motor works in conjunction with an APC 11X5.5EP propeller. A 10,000 mAh Lipo 4-cell 20 C battery was used and this propulsion system provides a maximum cruse time of approximately 40 min at an airspeed of 18 m/s.

### 2.4. Navigation System

The main component of the navigation system is the Pixhawk flight controller running the free ArduPilot Plane firmware, equipped with GPS and compass kit, airspeed sensor and a sonar for measuring the height below 7 m. The airplane with this navigation system can conduct a fully autonomous mission, including auto take-off, cruise via waypoints, return to home position and auto landing, with enhanced fail-safe protection.

### 2.5. GCS and Data Link

The GCS works via a data link that enables the researcher to monitor or interfere with the UAV during an auto mission. Mission Planner, an open-source ground station application compatible with Windows, was installed on the GCS laptop for mission design and monitoring. An HKPilot 433 Mhz 500 Mw radio transmitter and receiver was installed on the GCS laptop, along with a Pixhawk flight controller. An auto antenna tracker (AAT) worked in conjunction with a 9 dBi patch antenna to provide a reliable data link within a 5-km range.

### 2.6. Post-Imaging Processing and Video Transmission System

**Figure 4:**
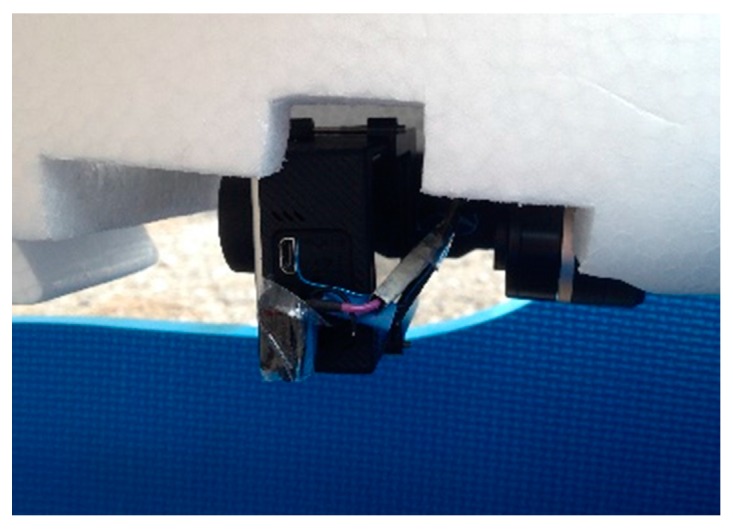
GoPro HERO 4 attached to the camera gimbal.

The UAV system is designed with a fixed-wing aircraft flying at airspeeds ranging from 15 to 25 m/s for quicker response times on SAR missions. The ground speed may reach 40 m/s in extreme weather conditions. A GoPro HERO 4 was installed in the vehicle after considering the balance between its weight and image quality capabilities. In a searching and mapping mission, the aerial image always faces the ground. During flight, some actions such as rolling, pitching or other unexpected vibrations can disrupt the camera’s stability, which may lead to unclear video. A Mini 2D camera gimbal produced by Feiyu Tech Co., Ltd. (Guilin, China), powered by two brushless motors, was used to stabilize the camera (Figure 4). The camera (GoPro HERO 4, GoPro, Inc., San Mateo, CA, USA) was set to video mode with a 1920 × 1080 pixel resolution in a narrow field of view (FOV) at 25 frames per second. During the flight, an analog image signal is sent to an on-screen display (OSD) and video transmitter. With a frequency of 5.8 GHz, the aerial video can be visualized by GCS in real-time as the high-resolution video is rerecorded for use during post-processing.

### 2.7. On-Board, Real-Time Imaging Process and Transmission System

**Figure 5:**
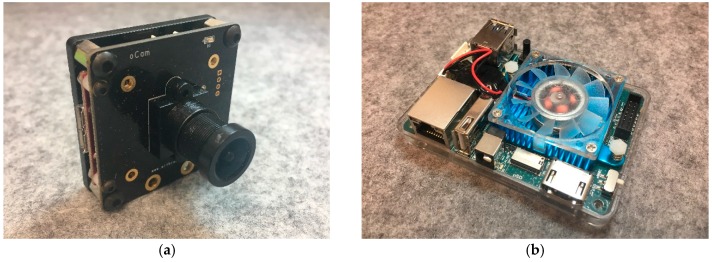
(**a**) oCam [25] and (**b**) Odroid XU4.

A real-time imaging process and transmission system was setup on the UAV. The “oCam,” (shows in Figure 5) a 5-mega pixel charge-coupled device (CCD) camera was chosen as the image source for the on-board target identification system. The focal length of the camera is 3.6 mm and it has a field of view of 65°. It weighs 37 g and has a 1920 × 1080 pixel resolution with 30 frames per second. The development of the on-board image processing was based on the Odroid XU4 (Hardkernel co., Ltd., GyeongGi, South Korea) (Figure 5b), which is a light, small, powerful computing device equipped with a 2-GHz core CPU and 2 Gbyte LPDDR3 Random-Access Memory (RAM). It also provides USB 3.0 interfaces that increase transfer speeds for high-resolution images. The Odroid XU4 used on the UAV in this system runs Ubuntu 14.04. The details of the algorithm and implementation will be discussed in Section 3. The Odroid board was connected to a 4th Generation (4G) cellular network via a HUAWEI (Shenzhen, China) E3372 USB dongle. Once the target is identified by the Odroid XU4, that particular image is transmitted through the 4G cellular network to the GCS.

## 3. Algorithm for and Implementation of Target Identification and Mapping

The target identification program was implemented using an on-board micro-computer (Odroid XU4,) and the ground control station. The program can automatically identify and report cars, people and other specific targets.

### 3.1. Target Identification Algorithm

**Figure 6:**
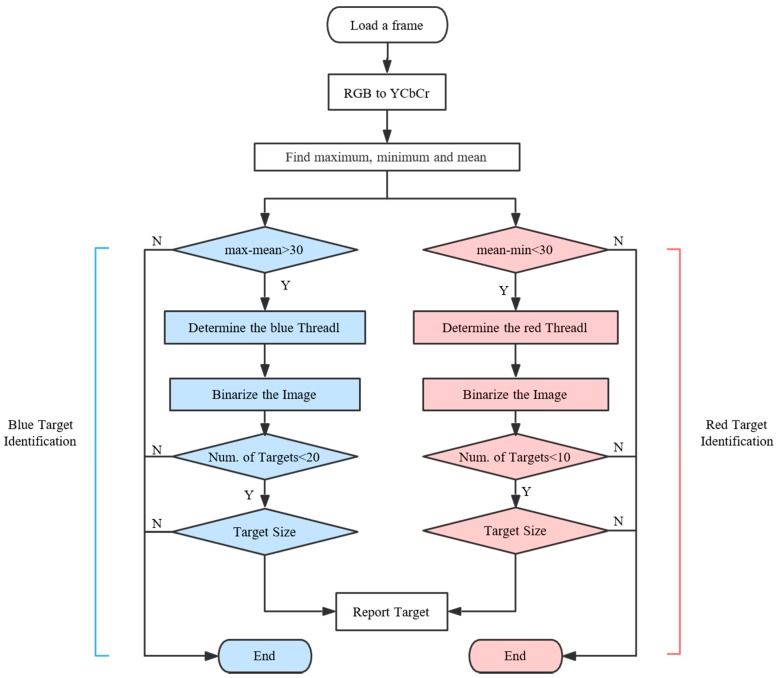
Flowchart of the identification algorithm.

The mission is to find victims who need to be rescued, crashed cars or aircraft. The algorithm approaches these reconnaissance problems by using the color signature. These targets create a good contrast with the backgrounds due to their artificial colors. Figure 6 shows the flowchart of the reconnaissance algorithm. The aerial images are in YUV rather than RGB color space to identify the color signatures [26]. This progress can be achieved by calling back the function provided by OpenCV libraries. Both blue and red signatures are examined.

The crucial step of the algorithm is to find an appropriate value of Threadl. A self-adapting method was applied to the reconnaissance program. The identification included the following steps.

*Step 1*: Read the blue and red chrominance values (Cb and Cr layers) of the image, and determine the maximum, minimum and mean values of the chrominance matrix. These values are then used to adapt the threshold.
*Step 2*: Distinguish whether existing objects are in great contrast. The distinction is processed by comparing the maximum/minimum and mean values of the chrominance. Introducing this step improves the efficiency with which the aerial video is processed, because the relevant identification is skipped if the criteria are not met. The criteria are expressed in Equation (1):
  (1) $$\begin{array}{l} \max-\mathrm{mean}>30\\ \mathrm{mean}-\min<30 \end{array}$$
*Step 3*: Determine the appropriate value of the threshold, which is determined by Equation (2), where the threshold with subscripts b and r donate blue and red, respectively. $K_{s}$ is the sensitivity factor, and the program becomes more sensitive as it increases. $K_{s}$ also changes with different cameras, and was set as 0.1 for the GoPro HERO 4 and 0.15 for the oCam in this study.
  (2) $$\begin{array}{l} Threadl_{b}=\max-(\max-\mathrm{mean})*K_{s}\\ Threadl_{r}=(\mathrm{mean}-\min)*K_{s}+\min \end{array}$$
*Step 4*: Binarize the image with the threshold.
  (3) $$f(p)=\left\{ \begin{array}{r} 0;(p<Threadl)\\ 255;(p>Threadl) \end{array}\right.$$
  where 0 represents the black color and 255 represents the white color.
*Step 5*: Examine the number of targets and their sizes. The results are abandoned if there are too many targets (over 20) in a single image because such results are typically caused by noise at the flight height of 80 m. The amount criterion is used because it is rare for a UAV to capture over 20 victims or cars in a single image in the wilderness. When examining the size of the targets, the results are abandoned if the suspected target only has a few or too many pixels. The criterion for the number of pixels is determined by the height of the UAV and the size of the target.
*Step 6*: The targets are marked with blue or red circles on the original image and reported to the GCS.

**Figure 7:**
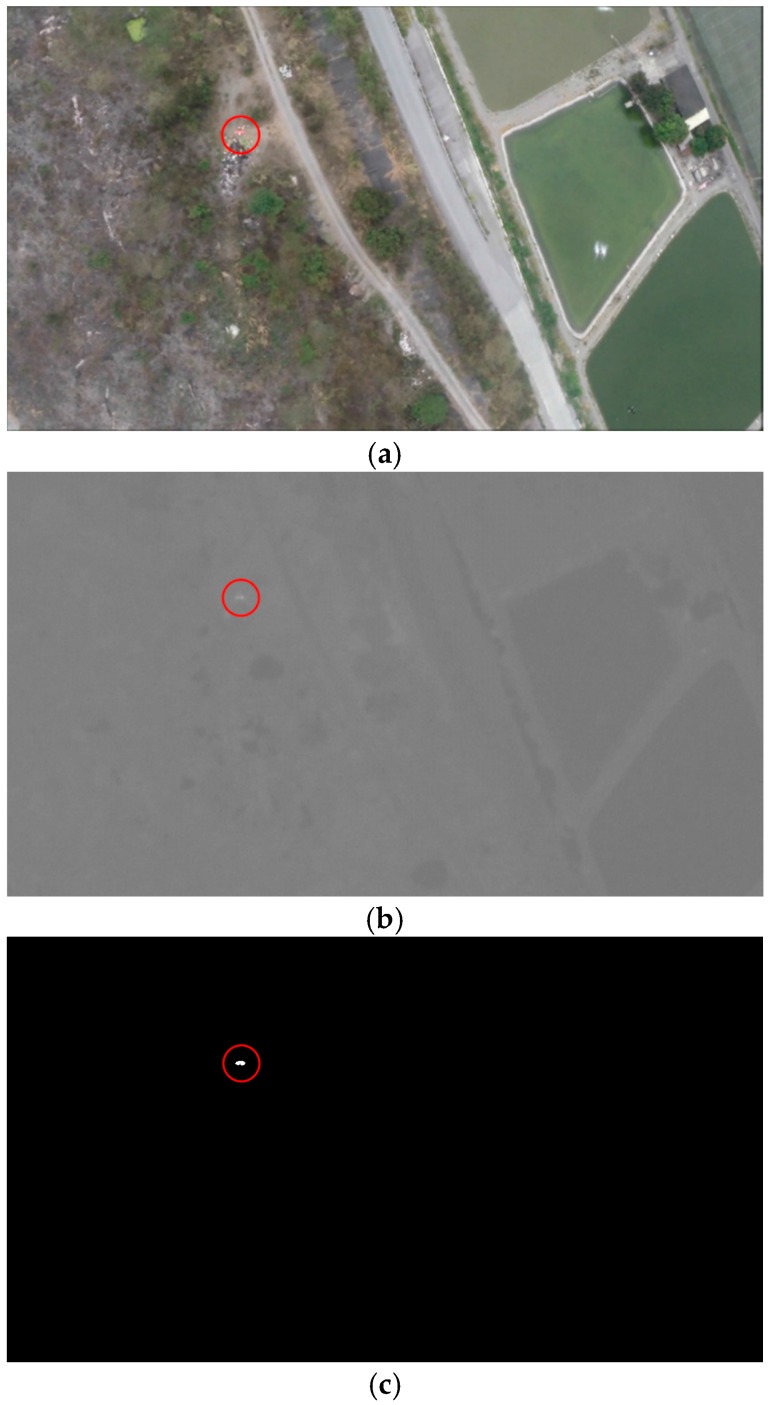
(**a**) The original image with red target in RGB color space; (**b**) the Cr layer of the YCbCr color space and (**c**) the binarized image with threshold.

Figure 7 demonstrates a test of the target identification algorithm using an aerial image with a tiny red target. Figure 7a is the original image captured from the aerial video with the target circled for easy identification. The Cr data were loaded for red color, as shown in Figure 7b. Figure 7c shows the results of the binarized image with a threshold of 0.44 (the white spot in the upper left quadrant).

### 3.2. On-Board Target Identification Implementation

**Figure 8:**
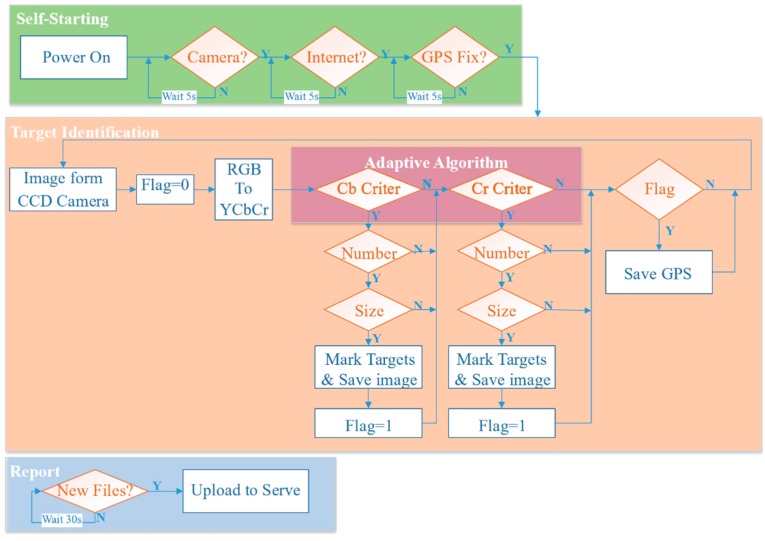
Flowchart of the on-board target identification system.

Before developing the on-board system for identifying targets, the method used to report the targets and their locations to the GCS must be determined. Considering all of the subsystems on the vehicle and the frequencies used for the data link (433 MHz), live video transmission (5.8 GHz) and remote controller (2.4 GHz), the on-board target identification system is designed to connect to the base station of a cellular network, 800–900 MHz in the proposed testing area (Hong Kong and Taiwan). The results are then uploaded to the Dropbox server. Consequently, the on-board target identification system consists of four modules: Odroid as the core hardware, an oCam CCD camera, a GPS module and a dongle that connects to the 4G cellular network and provides it for the Odroid. The workflow of the on-board target identification system, designed as shown in Figure 8, includes three functions: Self-starting, identification and target reporting.

The self-starting is achieved via a Linux shell script. The program runs automatically when Odroid is powered on. The statuses of the camera, the Internet and the GPS module are checked. After successfully connecting all of the modules, the identification program runs on a loop until the Odroid is powered off. The identification program usually conducts four frames in a second.

During the flight, the GPS coordinates of the aircraft are directly treated as the location of the targets, because the rapid report is preferable to taking the time to get a highly accurate report during flight. The accurate locations of the targets are discovered post-flight using the high-resolution aerial video taken by the GoPro camera.

**Figure 9:**
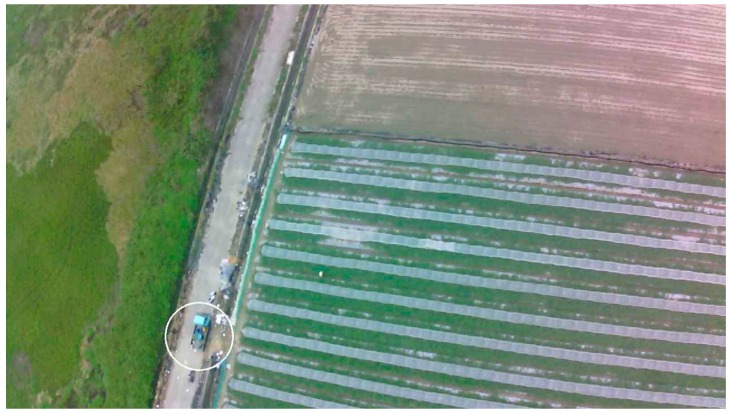
A blue truck reported by the on-board target identification system, marked by the identification program with a white circle.

When reporting, the system scans the resulting files every 30 s and packs the new results, which are uploaded as a package instead of as frames to limit time consumption, because the Dropbox server requires verification for each file. The testing results show that uploading a package every 30 s is faster than uploading frame by frame. The reporting results include the images of the marked target and a text file of the GPS coordinates. These files are then stored in an external SD card that allows the GCS to quickly check the results post-flight. Figure 9 shows a truck reported by the on-board target identification system.

### 3.3. Post-Target Identification Implementation via Aerial Video and Flight Log

Post-target identification is conducted using the high-resolution aerial video taken by the GoPro camera and stored in the SD card, and the flight data log from the flight controller to capture all possible targets to be rescued and obtain their accurate locations. In this section, the technical details of post-target identification are discussed.

#### 3.3.1. Target Identification

The altitude of the flight path is carefully determined during the flight tests via the inertial-measurement unit and GPS data in the flight controller. Any targets coated with artificial colors of or larger than the estimated image size (15×15 pixels), calculated according to the height of the UAV and the target’s physical dimensions, should be reported.

**Figure 10:**
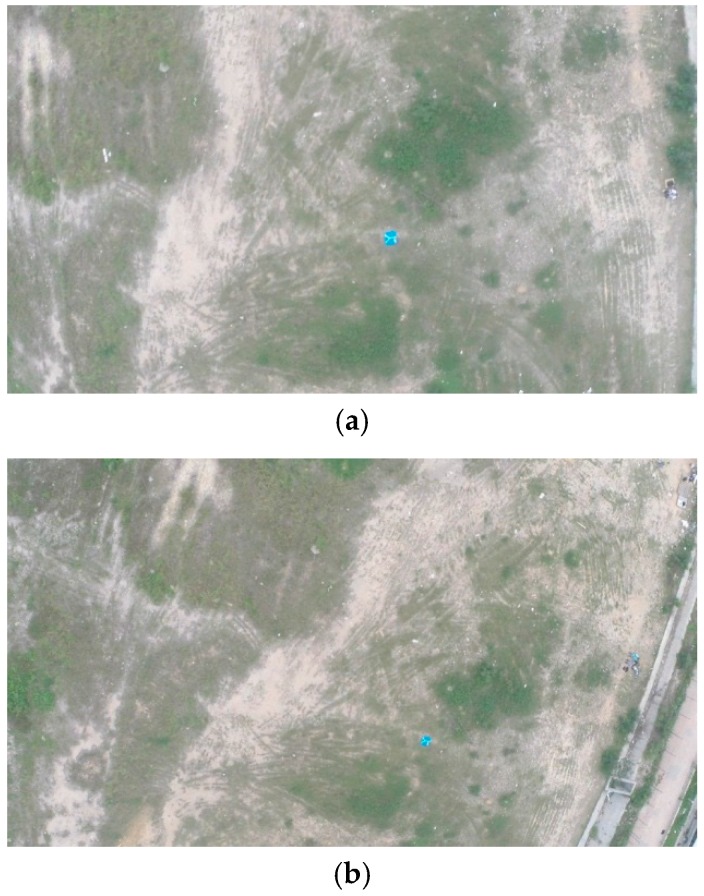

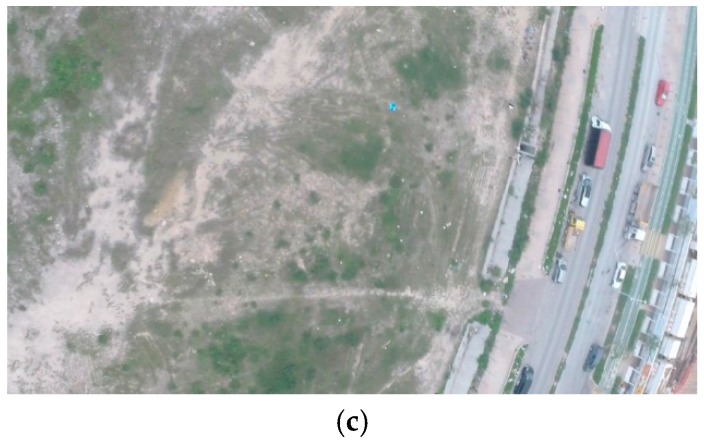
The results of altitude tests with the vehicle cruising at (**a**) 50 m; (**b**) 80 m and (**c**) 100 m.

Figure 10 shows an aerial image of a 0.8 m×0.8 m blue board with a letter ‘Y’ on it from flight heights of 50 m, 80 m and 100 m. The height of the flight path for the later field test was determined to be lower than 80 m accordingly, otherwise, the targets would only be several pixels in the image and might be treated as noise.

**Figure 11:**
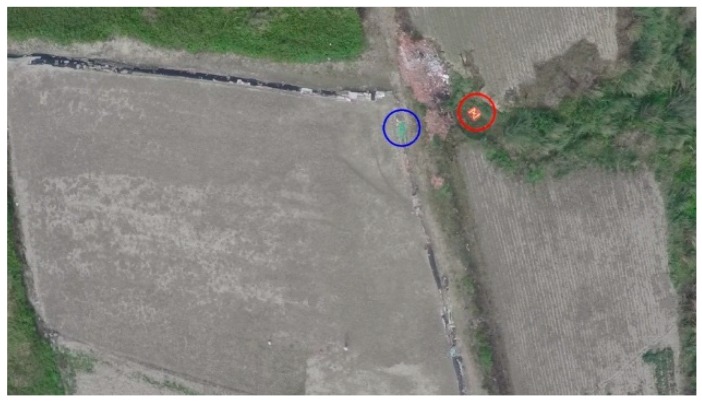
A red target board and a green agricultural net reported by the post-identification program, with both the red and blue targets marked with circles in corresponding colors.

The main loop of the post-identification program was developed in the OPENCV environment. Similar to on-board target identification, the post-identification program loads the aerial video file and runs the algorithm in a loop with each frame. The targets are marked for the GCS operator, who engages in efficient confirmation. The flight data log and the aerial video are simultaneously synchronized to determine the reference frame number and reference shutter time. The technical details of this step are discussed in Section 3.3.2. The target image is saved as a JPEG file and named with its frame number. Figure 11 shows a red target board and a green agricultural net reported by the post-identification program. This JPEG file is sent to the GPS transformation program discussed in Section 3.3.3 to better position the target.

To determine the image’s frame number, we assume that the GoPro HERO 4 camera records the video with a fixed frame rate of 25 frames per second (FPS) in this study. Thus, the time interval (TI) of the target frame **F** in the aerial video and the reference frame can be determined by

(4) TI=(Frame Number−Reference Frame No.)×40 ms

and the GPS time of **F** is

(5) GPSTime=Reference GPS Time+TI

where the Reference Frame No. and Reference GPS Time are determined during synchronization, as discussed in Section 3.3.2.

Once the GPS time of the target frame is determined, the altitude and GPS coordinates of the camera are determined. The yaw angle Ψ is recorded as part of the Attitude messages in the flight data log, and the corresponding Attitude message can be searched via GPS time. The update frequencies of the Attitude messages come from an inertial-measurement unit IMU sensor, and the GPS messages are different. These two types of messages cannot be recorded simultaneously due to the control logic of the flight board. However, the updating frequency of the Attitude message is much higher than that of the GPS messages, thus the attitude message that is closest to the GPS time is treated as the vehicle’s current attitude.

#### 3.3.2. Synchronization of the Flight Data and Aerial Video

During the flight, the aerial video and flight data are recorded by the GoPro HERO 4 camera and flight controller, respectively. It is crucial to synchronize the flight data and the aerial video to obtain the targets’ geo-information for the identification and mapping of the affected areas in a rescue mission.

**Figure 12:**
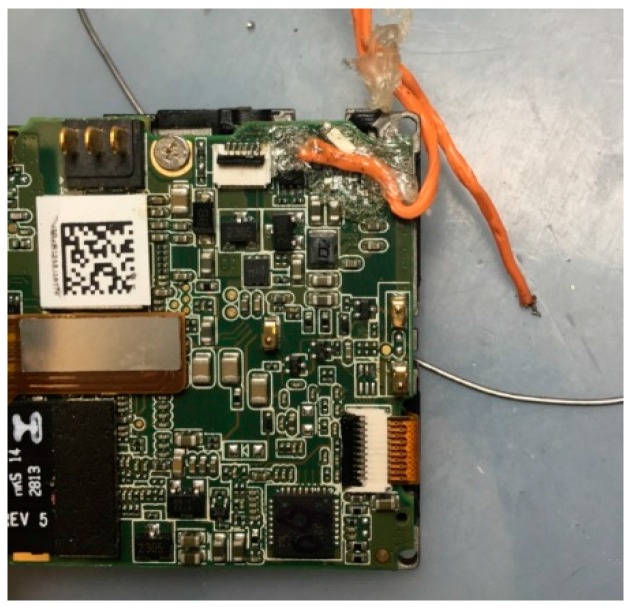
Modification of the GoPro buttons to PWM-controlled relay switch.

Camera trigger distance (DO_SET_CAM_TRIGG_DIST), a camera control command provided by ArduPlane firmware, was introduced to synchronize the aerial video and the flight data log. DO_SET_CAM_TRIGG_DIST sets the distance in meters between camera triggers, and the flight control board logs the camera messages, including GPS time, GPS location and aircraft altitude when the camera is triggered. Compared with commercial quad-copters, fixed-wing UAVs fly at higher airspeeds. The time interval between two consecutive images should be small enough to meet the overlapping requirement for further mapping. However, the normal GoPro HERO 4 cannot achieve continuous photo capturing at a high frequency (5 Hz or 10 Hz) for longer than 30 s [27]. Thus, the GoPro was set to work in video recording mode with a frame rate of 25 FPS. The mode and shutter buttons were modified with a pulse width modulation (PWM)-controlled relay switch, as shown in Figure 12, so that the camera can be controlled by the flight controller. The shutter and its duration are configured in the flight controller.

The camera trigger distance can be set to any distance that will not affect the GoPro’s video recording. A high-frequency photo capturing command will lead to video file damage. In this study, the flight controller sends a PWM signal to trigger the camera and record the shutter times and positions of the camera messages. However, the Pixhawk records the time that the control signal is sent out, and there is a delay between the image’s recorded time and its real shutter time. This shutter delay was measured to be 40 ms and was introduced to the synchronization process.

**Figure 13:**
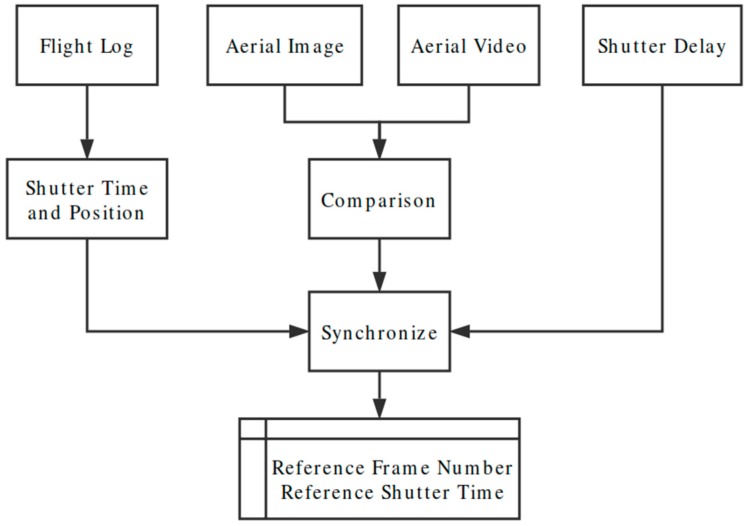
Flowchart for the synchronization of the aerial video and the flight data log.

**Figure 14:**
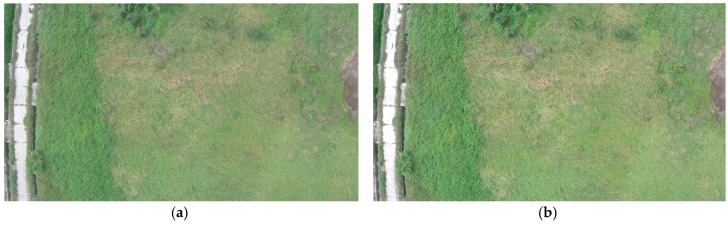
Comparison results in synchronization process (**a**) the original photo taken by Gopro camera and (**b**) video frame captured by synchronization program.

The synchronization process shown in Figure 13 is conducted after the flight. The synchronization process shown in Figure 13 is conducted after the flight. The comparison process started with reading the aerial video and the photograph saved in GoPro’s SD card. The original captured photo was resized to 1920×1080 pixels because the GoPro photograph was of a nonstandard size of 2016×1128 pixels. During the comparison process, both the video frames and photograph were treated as a matrix with a size of 1920×1080×3, where the number 3 denotes the 3 layers of RGB color space. The difference ε between the video frame and the photo was determined by the mean-square deviation value of $\left(Matrix_{photo}-Matrix_{frame}\right)$. The video frame with minimum value of ε was considered the same as the original aerial photo (Figure 14) and the number of this video frame was recorded as the Reference Frame No (RFN). The recorded GPS time of sending the aerial photo triggering command was named as the Reference GPS time (RGT). Considering the above-mentioned 40 ms delay between sending out the command and capturing the photo the frame at RFN was taken at the time of (RGT + 40 ms delay time). Therefore, the video is combined with the flight log.

#### 3.3.3. GPS Transformation to Locate Targets

Once a target with its current aircraft position is reported to the GCS, an in-house MatLab locating program is used to report the target’s GPS coordinates. In this study, the position of the aircraft is assumed to be at the center of the image, because the GPS module is placed above the camera.

**Figure 15:**
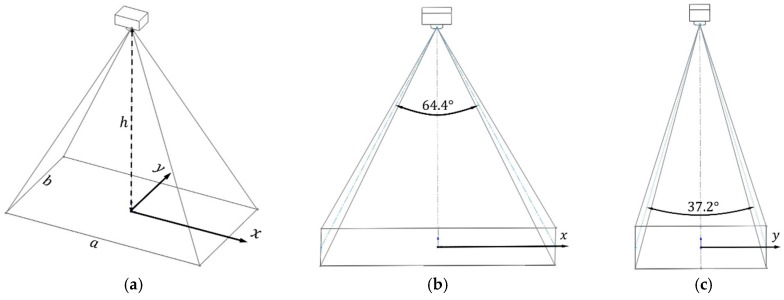
Camera and world coordinates.

The coverage of an image can be estimated using the camera’s field of view (FOV) [28], as shown in Figure 15. The distances in the x and y directions are estimated using Equation (6).

(6) $$a=\frac{2h}{\cos\left(\frac{{\mathrm{FOV}}_{X}}{2}\right)}$$

$$b=\frac{2h}{\cos\left(\frac{{\mathrm{FOV}}_{Y}}{2}\right)}$$

The resolution of the video frame is set to be 1920×1080 pixels. The scale between the distance and pixels is assumed to be a linear relationship, and is presented in Equation (7) as:

(7) $$scale_{x}=\frac{a}{1920}=\frac{2h}{1920\left(\frac{{\mathrm{FOV}}_{X}}{2}\right)}$$

$$scale_{y}=\frac{b}{1080}=\frac{2h}{1080\left(\frac{{\mathrm{FOV}}_{Y}}{2}\right)}$$

**Figure 16:**
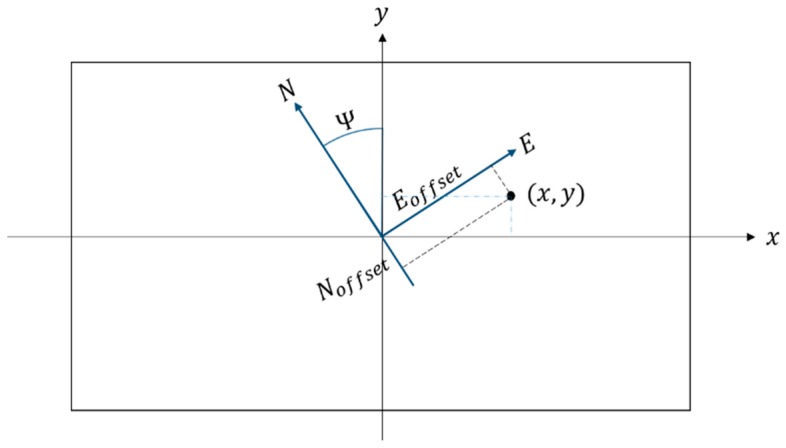
Coordinates of the camera and world frames.

As Figure 16 shows, a target is assumed to be located on the (x,y) pixel in the photo, and the offset of the target from the center of the picture is

(8) $$offset_{target}=\left[\begin{array}{c} scale_{x}\cdot x\\ scale_{y}\cdot y \end{array}\right]\left(m\right)$$

For the transformation of a north-east (NE) world-to-camera frame with the angle of the Ψ, the rotation matrix is defined as

(9) $$R_{W}^{C}=\left[\begin{array}{cc} \cos\left(\Psi \right) & -\sin\left(\Psi \right)\\ \sin\left(\Psi \right) & \cos\left(\Psi \right) \end{array}\right]$$

where Ψ is the yaw angel of the aircraft. Thus, the position offset in the world frame can be solved with

(10) $$P={R_{W}^{C}}^{T}offset_{target}=\left[\begin{array}{c} P_{E}\\ P_{N} \end{array}\right]$$

Therefore, the target’s GPS coordinates can be determined using

(11) $$GPS_{target}=GPS_{cam}+\left[\begin{array}{c} P_{E}/f_{x}\\ P_{N}/f_{y} \end{array}\right]$$

where $f_{x}$ and $f_{y}$ denote the distances represented by one degree of longitude and latitude, respectively.

**Figure 17:**
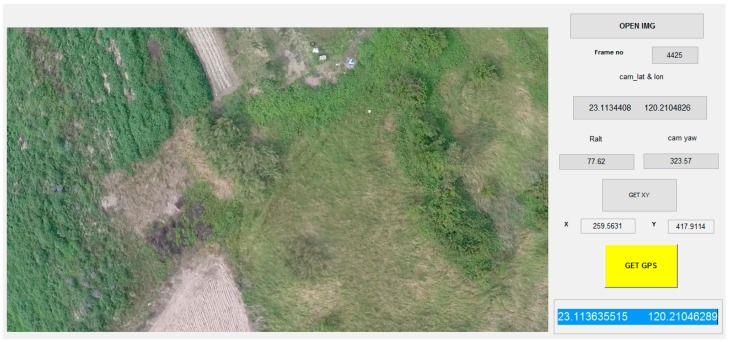
Graphical user interface for the GPS transformation that allows end users to access a target’s GPS coordinates using simple buttons.

A graphical user interface was designed and implemented in the MatLab environment to transform the coordinates with a simple ‘click and run’ function (Figure 17). The first step is opening the image containing the targets. The program automatically loads the necessary information for the image, including the frame number (also the image’s file name), current location, camera attitude and yaw angle of the plane. The second step is to click the ‘GET XY’ button and use the mouse to click the target in the image. The program shows the coordinates of the target in this image. Finally, clicking the ‘GET GPS’ button provides the GPS coordinates reported by the program.

### 3.4. Mapping the Searched Area

During rescue missions following landslides or floods, the terrain features can change significantly. After target identification, the local map must be re-built to guarantee the rescue team’s safety and shorten the rescue time. In this study, we provide a preliminary demonstration of a fixed-wing UAV used to assist in post-disaster surveillance. Mapping algorithms are not discussed in this paper. The commercial software Pix4D was used to generate orthomosaic models and point clouds.

**Figure 18:**
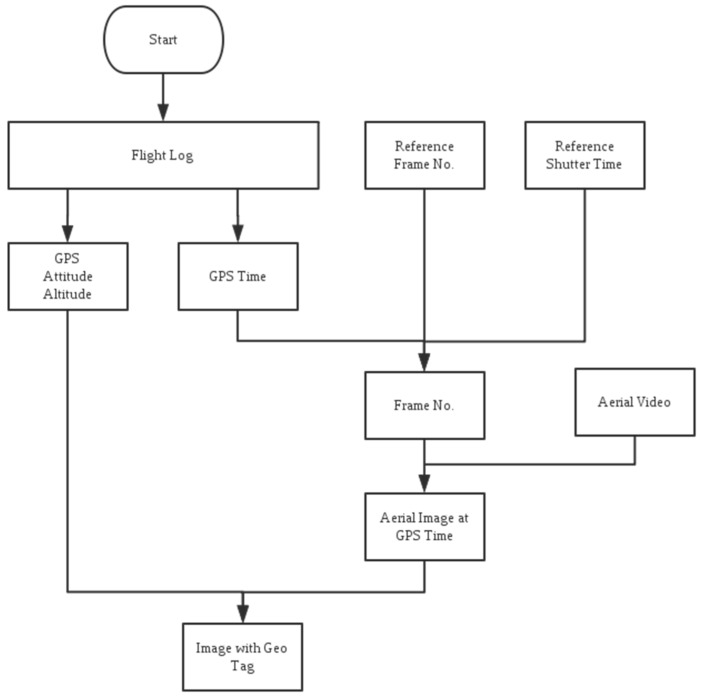
Flowchart of the mapping image capture program.

To map the disaster area, a set of aerial photos and their geo-information are applied to the commercial software, Pix4D. There should be at least 65% overlap between consecutive pictures, but aiming for 80% or higher is recommended. The distance between two flight paths should be smaller than a, and estimation Equation (6) can be found in Section 3.3.3. A mapping image capture program is shown in Figure 18.

The mapping image capture program starts with GPS messages from the flight data log with reference frame numbers and shutter times generated by the synchronization step discussed in Section 3.2. The program loads the GPS times of all of the GPS messages in the loop and calculates the corresponding frame number N in the aerial video, which equals

$$N=\frac{GPS\;Time-Reference\;GPS\;Time}{40\;\mathrm{ms}}+Reference\;Frame\;No.$$

Then, the mapping image capture program loads the Nth frame of the aerial video and saves it to the image file.

Once the mapping image capture program is complete, a series of photos and a text file containing the file names, longitude, latitude, altitude, roll, pitch and yaw are generated. The Pix4D then produces the orthomosaic model and point clouds using these two file types.

## 4. Blind Tests and Results

**Figure 19:**
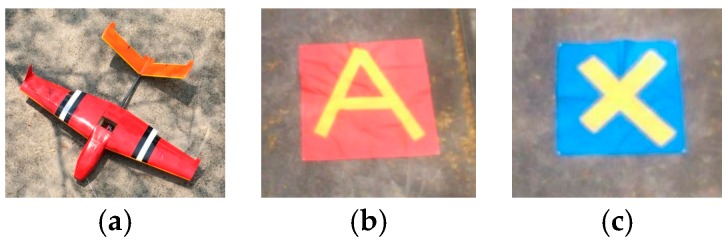
(**a**) The drone simulated a crashed airplane, (**b**) the 2 m×2 m blue or red target boards represented broken cars and (**c**) the 0.8 m×0.8 m blue or red targets boards represented injured people to be rescued.

To test the all-in-one camera-based target detection and positioning system, a blind field test was designed. A drone, a 2 m×2 m blue or red square board and a 0.8 m×0.8 m blue or red square board were used to simulate a crashed airplane, broken cars and injured people, respectively (Figure 19a–c).

The flight tests were conducted at two test sites, the International Model Aviation Center (22°24′58.1′′ N 114°02′35.4′′ E) of the Hong Kong Model Engineering Club, Ltd. in Yuen Long town, Hong Kong and the Zengwun River (23°7′18.03′′ N 120°13′53.86′′ E) in the Xigang District, of Tainan city, Taiwan. Given concerns with the limited flying area in Hong Kong, the preliminary in-sight tests were conducted in Hong Kong and the main blind out-of-sight tests were conducted in Taiwan. The flight test information is listed in Table 1. Only post-identification tests were conducted in Hong Kong. In Taiwan, no after-flight mapping was done for the first two tests (Tests 3 and 4).

**Figure 20:**
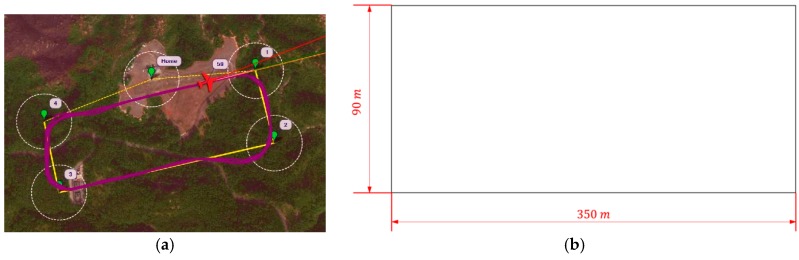

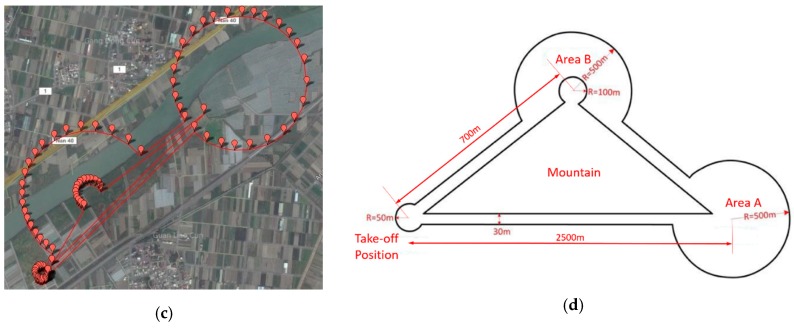
(**a**) Test route in Hong Kong; (**b**) schematics of the designed route in Hong Kong; (**c**) search areas A and B for blind tests in Taiwan and (**d**) schematics of the designed search route and areas in Taiwan.

Figure 20a,b shows the search site and its schematic in Hong Kong. The search path repeated the square route due to the limited flight area. The yellow path in Figure 20a is the designed mission path and the purple line indicates the real flight path of the vehicle. For the tests in Taiwan, there were two main search areas (A and B) along the bank of the Zengwun River in the Xigang District of Tainan city, Taiwan, as shown in Figure 20c. The schematics of the designed search route and areas are depicted in Figure 20d. The maximum communication distance was 3 km and the width of the flight corridor was 30 m. This width was intended to test the stability of the UAV and the geo-fencing function of the flight controller. If the UAV flies outside the corridor, it is considered to have crashed. After the flight performance tests, the UAV flew inside the corridor and was proven stable. An unknown number of targets were placed in search areas A and B by an independent volunteer before every test. The search team then conducted the field tests and tried to find the targets. The test results are discussed in the following sections.

### 4.1. Target Identification and Location

Post-target identification processing was conducted in all eight flight test to assess the identification algorithm. The post-identification program ran on a laptop equipped with Intel Core i5-2430M CPU and 8 Gb RAM. The testing results are shown in Table 2. Note that the post-identification program only missed two targets for all of the tests.

**Figure 21:**
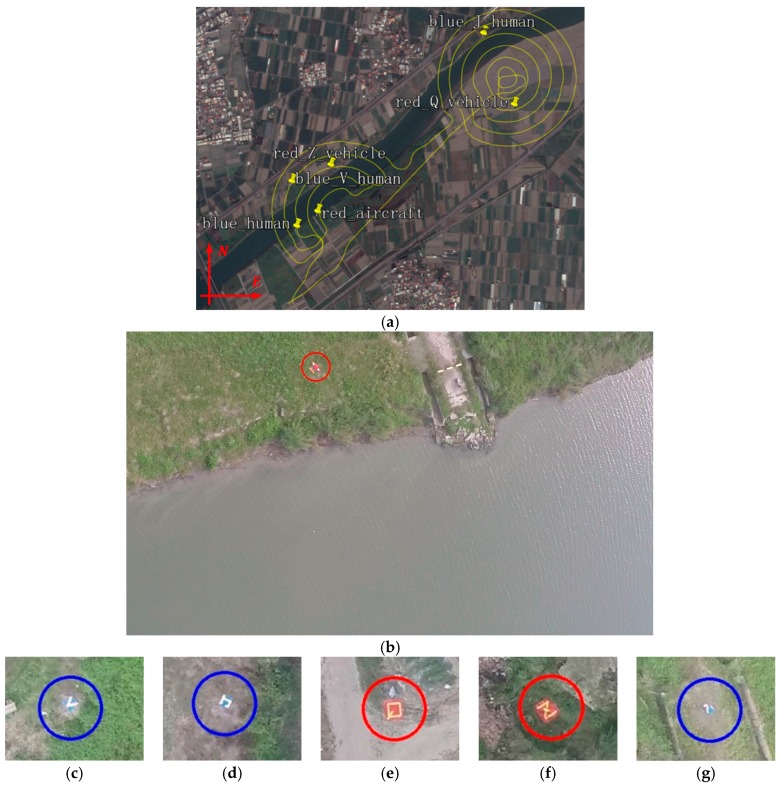
(**a**) The locations of six simulated targets; (**b**) the original image saved by the identification program with target drone; (**c**) designed target (blue board with letter V) represents an injured person; (**d**) designed target (blue board with letter J) represents an injured person; (**e**) designed target (red board with letter Q) represents a crashed car; (**f**) designed target (red board with letter Z) represents a crashed car and (**g**) designed target (small blue board) represents an injured person. The board was blown over by the wind.

Taking test 7 as an example, 6/6 targets were found by the identification system, as shown in Figure 21, including a crashed aircraft, two crashed cars and three injured people. Note that in Figure 21g the target board, representing the injured people, was folded by gusts of wind to the extent that it is barely recognizable. Nevertheless, the identification system still reported this target, confirming its reliability. The locating error of 5 targets was less than 15 m as shown in Table 3 (having met the requirements discussed in Section 1). The targets and their locations were reported in 15 min.

**Figure 22:**
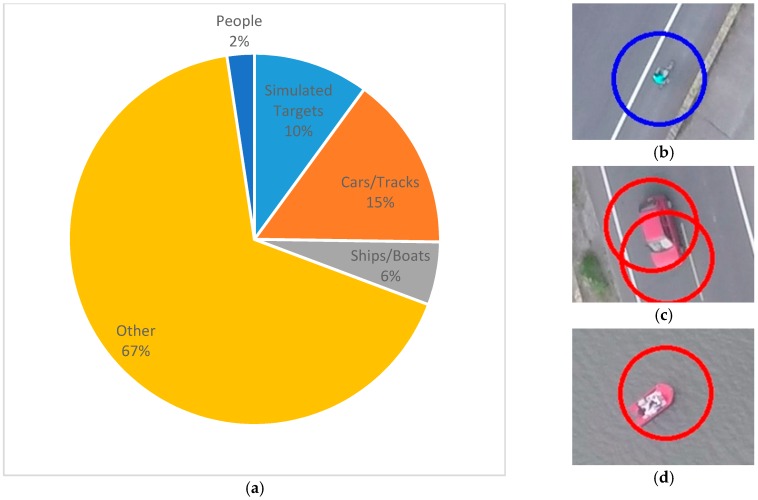
(**a**) Composition of reporting targets; (**b**) a person on the road; (**c**) a red car and (**d**) a red boat reported by the identification program.

**Figure 23:**
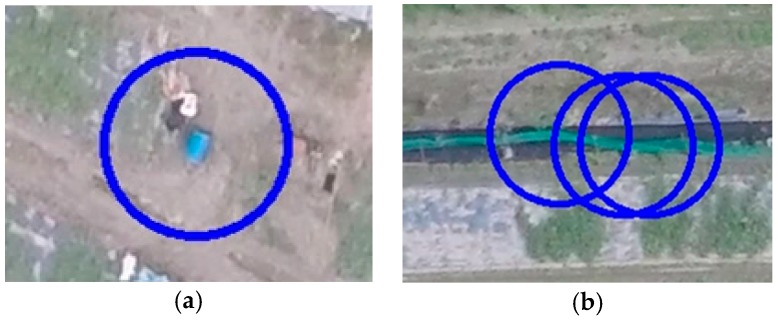
The other reporting targets: (**a**) A blue bucket and (**b**) green nets.

In addition to the designed targets, the identification program reported real cars/tracks, people, boats and other objects. The percentages of each type of target are shown in Figure 22. The large amount of other targets is due to the nature of the search area. The testing site is a large area of cropland near a river, and the local farmers use a type of fertilizer that is stored in blue buckets and they use green nets to fence in their crops. These two item types were reported, as shown in Figure 23. However, these results can be quickly sifted through by the GCS operator. The identification program still reduces the operator’s work load, and the search mission was successfully completed in 40 min, beginning when the UAV took off and ending when all of the targets had been reported.

**Figure 24:**
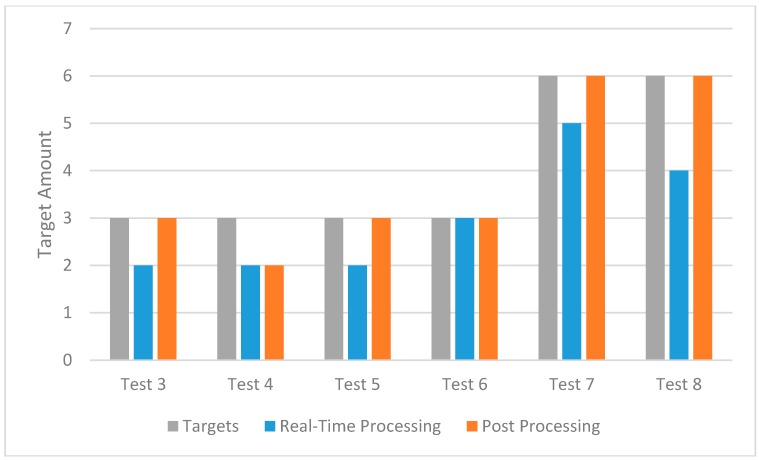
Target identification results of real-time processing and post-processing.

In tests 3–8, both on-board real-time processing and post-processing were conducted and the results are shown in Figure 24. Note that the performance of the post-target identification is better than that of real-time onboard target identification, due to the higher resolution of the image source. Nevertheless, the on-board target identification system still reported more than 60% of the targets and provided an efficient real-time supplementary tool for the all-in-one rescue mission. A future study will be conducted to improve the success rates of on-board target identification systems.

### 4.2. Mapping

**Figure 25:**
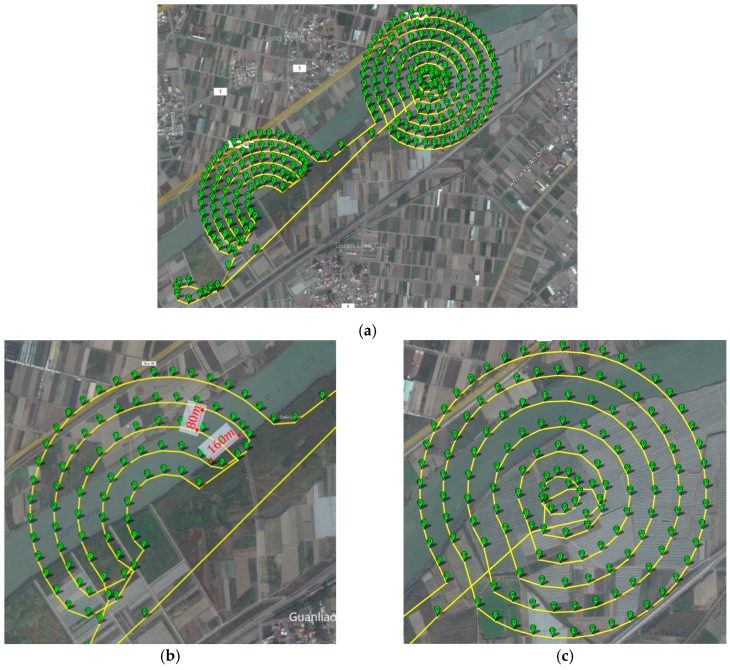
(**a**) Overall flight plan for the search mission, (**b**) flight plan for search area B (the turning diameter reaches 160 m to ensure the flight performance while the distance between the two flight paths remains 80 m, guaranteeing full coverage and overlap) and (**c**) flight plan for search area A.

To cover the whole search area, the flight plan was designed as shown in Figure 25. The distance between 2 adjacent flight paths is 80 m. The total distance of flight plan is 20.5 km with a flight time of 18 min. The turning radius of the UAV was calculated, and it is 50 m for bank angles no larger than 35°. Thus, as shown in Figure 25b, the flight plan was designed with a 160-m turning diameter while the gap between the two flight paths remained 80 m to ensure overlapping and complete coverage.

**Figure 26:**
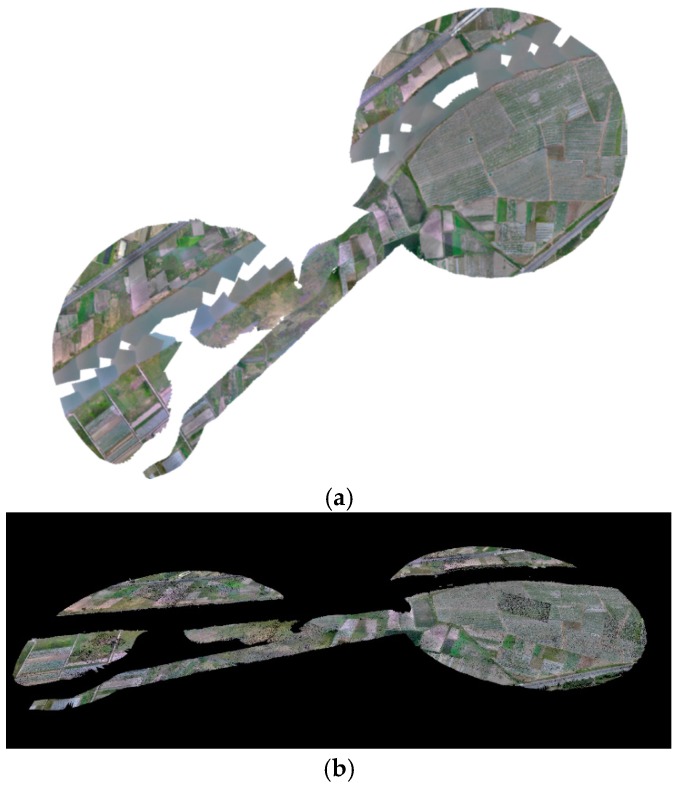
(**a**) Orthomosaic model of the testing area and (**b**) point clouds of the search area.

After the flight, the mapping image capture program developed in this study was applied to capture the images from the high-resolution video and process the flight data log. A total of 2200 photos were generated and applied to Pix4D, and the resulting orthomosaic model and point clouds are shown in Figure 26. The missing part is due to the strong reflection on the water’s surface resulting in mismatched features.

## 5. Conclusions

In this study, a UAV system was developed, and its ability to assist in SAR missions after disasters was demonstrated. The UAV system is a data acquisition system equipped with various sensors to realize searching and geo-information acquisition in a single flight. The system can reduce the cost of large-scale searches, improve the efficiency and reduce end-users’ workloads.

In this paper, we presented a target identification algorithm with a self-adapting threshold that can be applied to a UAV system. Based on this algorithm, a set of programs was developed and tested in a simulated search mission. The test results demonstrated the reliability and efficiency of this new UAV system.

A further study will be conducted to improve the image processing in both onboard and post target identification, focusing on reducing the unexpected reporting targets. A proposed optimization method is to add an extra filtration process to the GCS to further identify the shape of the targets. This proposed method will not increase the computational time of the onboard device significantly. It is a simple but effective method concerning the limited CPU capability of an on-board processor. Generally speaking, most commercial software is too comprehensive to be used in the on-board device. Notably, the limitation of the computing power becomes a minor consideration during post-processing since powerful computing devices can be used at this stage. To evaluate and improve the performance of targets’ identification algorithm in post-processing, further study will be conducted, including the application of the parallel computing technology and comparison with the advanced commercial software.

In this study, the scales of the camera and world coordinates were assumed to be linear. This assumption can result in target location errors. We tried to reduce the error by selecting the image with the target near the image center. Although the error of the current system is acceptable for a search mission, we will conduct a further study to improve the location accuracy. Lidar will be installed to replace the sonar, and more accurate relative vehicle height will be provided for auto-landing. Also, in the future, the vehicle will be further integrated to realize the ‘Ready-to-Fly’ stage for quick responses in real applications.

## Figures and Tables

**Table 1:** Basic information for flight tests.

Flight Test	Test Site	Flight Time (min)	Testing Function		
			Real-Time Identification	Post-Identification	Mapping
Test 1	Hong Kong	15:36	×	√	×
Test 2	Hong Kong	3:05	×	√	×
Test 3	Taiwan	13:23	√	√	×
Test 4	Taiwan	17:41	√	√	×
Test 5	Taiwan	17:26	√	√	√
Test 6	Taiwan	16:08	√	√	√
Test 7	Taiwan	16:23	√	√	√
Test 8	Taiwan	17:56	√	√	√

**Table 2:** Post-target identification results.

Flight Test	Resolution	Flying Altitude	Flight Time (min)	Targets	Identified Targets	Total Post-Target Identification Time (min)
Test 1	1920 × 1080	80	15:36	3	2	11:08.6
Test 2	1920 × 1080	80	3:05	2	2	02:46.3
Test 3	1920 × 1080	80	13:23	3	3	12:57.1
Test 4	1920 × 1080	80	17:41	3	2	14:04.6
Test 5	1920 × 1080	80	17:26	3	3	13:23.9
Test 6	1920 × 1080	80	16:08	3	3	11:45.1
Test 7	1920 × 1080	80	16:23	6	6	12:16.9
Test 8	1920 × 1080	75	17:56	6	6	14:18.3

**Table 3:** Locating results of flight test 7.

Target	Red Z	Red Plane	Blue I	Blue V	Blue J	Red Q
Latitude (N)	23.114536°	23.111577°	23.110889°	23.113637°	23.122189°	23.117840°
Longitude (E)	120.213111°	120.211898°	120.210819°	120.210463°	120.223206°	120.225225°
Error	2.8 m	13.9 m	1.6 m	0.8 m	11.3 m	4.8 m

## Supplementary Materials

The following is available online at https://www.youtube.com/watch?v=19_-RyPp93M. **Video S1:** A Camera-Based Target Detection and Positioning System for Wilderness Search and Rescue using a UAV. https://github.com/jingego/UAS_system/tree/master/Image%20Processing. **Source Code 1:** Matlab Code of targets identification. https://github.com/jingego/UAS_system/blob/master/Mapping_pre-process/CAM_clock_paper_version.m. **Source Code 2:** MatLab Code of synchronization.

## Abbreviations

The following abbreviations are used in this manuscript:

UAV:	Unmanned Aerial Vehicle
SAR:	Search and Rescue
GCS:	Ground Control System
AAT:	Auto Antenna Tracker
OSD:	On Screen Display
FOV:	Field of view
FPS:	Frame Per Second
